# KRAS Mutation Status Is Not a Predictor for Tumor Response and Survival in Rectal Cancer Patients Who Received Preoperative Radiotherapy With 5-Fluoropyrimidine Followed by Curative Surgery

**DOI:** 10.1097/MD.0000000000001284

**Published:** 2015-08-07

**Authors:** Jeong Won Lee, Jong Hoon Lee, Byoung Yong Shim, Sung Hwan Kim, Mi-Joo Chung, Bong-Hyeon Kye, Hyung Jin Kim, Hyeon Min Cho, Hong Seok Jang

**Affiliations:** From the Department of Radiation Oncology, Seoul St. Mary's Hospital (JWL, HSJ); Department of Radiation Oncology (JHL, SHK, MJC); Department of Medical Oncology (BYS); Department of Colorectal Surgery, St. Vincent's Hospital, College of Medicine, The Catholic University of Korea, Seoul, Korea (B-HK, HJK, HMC).

## Abstract

We evaluated the tumor response and survival according to the *KRAS* oncogene status in locally advanced rectal cancer. One hundred patients with locally advanced rectal cancer (cT3-4N0-2M0) received preoperative radiation of 50.4 Gy in 28 fractions with 5-fluorouracil and total mesorectal excision. Tumor DNA from each patient was obtained from pretreatment biopsy tissues. A Kirsten rat sarcoma viral oncogene homolog (KRAS) mutation was found in 26 (26%) of the 100 patients. Downstaging (ypT0-2N0M0) rates after preoperative chemoradiotheray were not statistically different between the wild-type and mutant-type KRAS groups (30.8% vs 27.0%, *P* = 0.715, respectively). After a median follow-up time of 34 months, there was no statistically significant difference in the 3-year relapse-free survival (82.2% vs 82.6%, *P* = 0.512) and overall survival (94.7% vs 92.3%, *P* = 0.249) rates between wild-type and mutant-type KRAS groups, respectively. The KRAS mutation status does not influence the tumor response to the radiotherapy and survival in locally advanced rectal cancer patients who received preoperative chemoradiotherapy and curative surgery.

## INTRODUCTION

For patients with locally advanced rectal cancer, preoperative chemoradiotherapy (CRT) followed by curative surgery is accepted as the standard treatment.^1^ This approach has shown to elicit a good tumor response and improve locoregional tumor control.^2^ Consequently, the National Comprehensive Cancer Network Guidelines recommend preoperative pelvic radiation of 50.4 Gy in 28 fractions with concurrent 5-fluorouracil (5-FU) and total mesorectal excision for locally advanced rectal cancer patients.^3^

There are a variety of reponses to preoperative CRT, ranging from minimal to excellent. Therefore, much research has reported about molecular markers like epidermal growth factor receptor (EGFR), vascular endothelial growth factor, phosphatase and tensin homolog, and tumor protein 53 as predictive markers for CRT,^4,5^ and the molecular markers have been applied to the treatment regimen.^6,7^ Among these molecules, the mutation of Kirsten RAS (*KRAS*) oncogene is a common event in carcinogenesis and is reported as a frequency of 30% to 40% in colorectal cancer.^8^ The Kirsten rat sarcoma viral oncogene homolog (KRAS) is a molecular transducer and an important component of the EGFR pathway.^9^ Several studies have searched for the prognostic significance of the KRAS mutation in colorectal cancer,^9–14^ but most of the literature is limited to the anti-EGFR therapy in metastatic colorectal cancer.^9–12^ When it comes to anti-EGFR therapy, some studies have identified the KRAS mutation as a predictor of unresponsiveness in metastatic colorectal cancer.^10,11^

However, few trials have investigated the *KRAS* oncogene status and clinical outcome in locally advanced rectal cancer patients who received 5-FU-based neoadjuvant CRT and curative surgery. Preclinical trials have suggested that the KRAS mutation would confer resistance to radiotherapy in rectal cancer,^15,16^ while there have been clinical analyses finding that the KRAS mutation did not predict the clinical efficacy of neoadjuvant CRT in rectal cancer.^4,8^ The role of the *KRAS* oncogene is unclear in rectal caner patients who received preoperative CRT and curative surgery,^17,18^ and clinical studies for this subject are scarce in the Asian population. Thus, we aimed to evaluate the clinical association between *KRAS* oncogene status and treatment outcome in locally advanced rectal cancer.

## METHODS AND MATERIALS

### Patients

**FIGURE 1:**
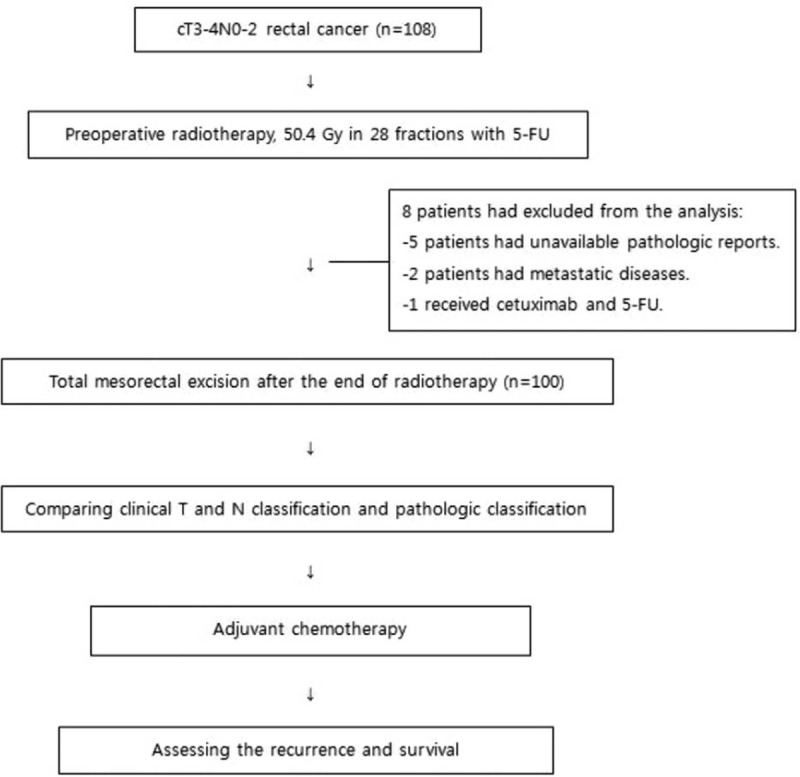
Flow chart of patient enrollment, chemoradiation, and surgery.

We retrospectively analyzed 108 patients with locally advanced rectal cancer who had received preoperative CRT and radical surgery. The patients were treated at our institutions between December 2008 and September 2013. Eligibility criteria included histologically confirmed rectal adenocarcinoma, clinical stage of cT3-4N0-2M0, and a tumor of the anal verge ≤8 cm. The exclusion criteria included patients with a history of malignancy other than rectal cancer, anti-EGFR therapy with preoperative radiotherapy, or distant metastasis at the time of diagnosis. Patients with cT1-2N0-2 were also excluded from this study. Of the 108 patients, 2 had evidence of distant metastases, 1 had radiotherapy with 5-FU and cetuximab (an anti-EGFR agent) to the pelvis, and the pathologic reports for the KRAS mutation in 5 patients were unavailable, all of whom were excluded from the study. The remaining 100 patients were finally analyzed (Figure 1). This study was approved by the Institutional Review Board of Catholic University of Korea (VC14RISI0197).

### Treatment

All patients underwent preoperative CRT. The radiotherapy consisted of 45 Gy in 25 fractions to the whole pelvis and 5.4 Gy in 3 fractions to the primary tumor with 3-field or 4-field box techniques. Concurrent chemotherapy was administered as bolus 5-FU (400 mg/m^2^/day) and leucovorin (20 mg/m^2^/day) infusion in the 1st and 5th week of radiotherapy. All analyzed patients received total mesorectal excision 4 to 8 weeks after the end of preoperative CRT. Adjuvant chemotherapy started 4 to 6 weeks after the radical surgery. Adjuvant chemotherapy consisted of 4 to 6 cycles of bolus 5-FU (500 mg/m^2^/day) on days 1 through 5, repeated monthly.

### Evaluation

Clinical staging examination before CRT consisted of digital rectal examination, complete blood count, liver and renal function test, level of carcinoembryonic antigen (CEA), video colonoscopy, chest and abdomen computed tomography, pelvic magnetic resonance imaging with or without endorectal ultrasonography (EUS), and positron emission tomography–computed tomography scans. The criterion for positive lymph node is defined as lymph node size of >5 mm on the magnetic resonance imaging and/or EUS. The pathologic tumor stage was categorized according to the tumor-node-metastasis classification of the American Joint Committee on Cancer Criteria, 7th edition.

Paraffin-embedded blocks of diagnostic biopsies and surgical specimens were cut in 5-μm sections. Sections were incubated in complete medium for 1 hour at room temperature with an EGFR rabbit monoclonal antibody (MU 207-UC; Biogenex, San Ramon, CA) at a dilution of 1:20. Immunohistochemical results of EGFR were evaluated according to extension and intensity. Extension was defined as the positive tumor cell percentage. EGFR was said to have positive staining when extension was 5% or more. When the extension was less than 5%, staining was considered negative. Tumor DNA from each patient was obtained from pretreatment biopsy tissues. Tumor cells were isolated using microdissection and genomic DNA was extracted. Standard polymerase chain reaction analysis was performed to detect specific mutations in KRAS (exons 2 and 3) using established primers.

After curative surgery, experienced colorectal pathologists evaluated the pathologic specimen. They assessed the histologic grade, presence of lymph node metastasis, response to CRT, and circumferential and distal rectal margins in the pathologic specimen. Tumor regression grade after CRT was classified according to the Dworak grading system.^19^ We defined pathologically complete response as total disappearance of a viable tumor after CRT. We compared preclinical and post-CRT pathological stages and defined downstaging as ypStage 0–I (ypT0-2N0M0).

### Statistical Analysis

The study was designed to identify whether KRAS mutation status is associated with tumor response to preoperative CRT and patient survival. Overall survival (OS) was defined as the time from the start of radiotherapy to death from any cause. Relapse-free survival (RFS) was defined as the time from the start of radiotherapy to any type of recurrence and death.

Survival distributions were calculated by the Kaplan–Meier method and compared using the log-rank test. The Chi-square test was used to compare the incidences of categorical variable, and the Student *t*-test was used to compare the means of continuous variables. Multivariate analysis using a logistic regression model was performed to determine associations between categorical variables and tumor response after CRT. All statistical tests were 2-sided, and *P*-values < 0.05 were considered statistically significant.

## RESULTS

Patient and tumor characteristics at baseline are shown in Table 1. All the patients received the prescribed radiotherapy and more than 90% of the included patients received chemotherapy as planned without delays. The study cohort consisted of 62 males and 38 females. The median age was 61 years (range, 27–80 years). KRAS mutation status is shown in Table 2. The KRAS mutation was found in 26 (26.0%) of 100 patients. KRAS mutations were detected at codon 12 (1 G12A, 2 G12C, 11 G12D, 2 G12S, and 4 G12V) in 20 cases and at codon 13 (all G13D) in 8 cases. Two patients had KRAS mutations which were located in the 2 sites simultaneously (G12C and G13D; G12D and G13D). Positive EGFR expression was observed in 71 (71%) of 100 patients.

**TABLE 1:**
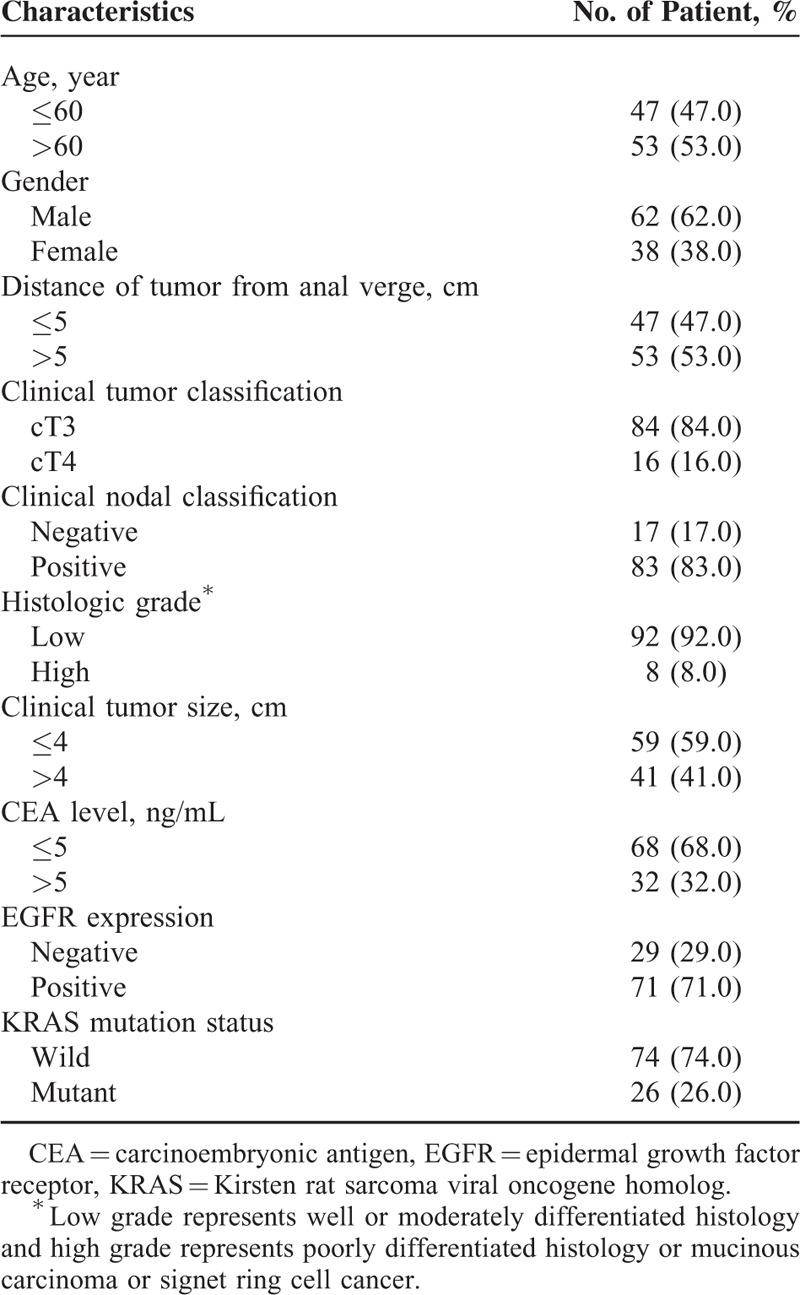
Patient and Tumor Characteristics (n = 100)

**TABLE 2:**
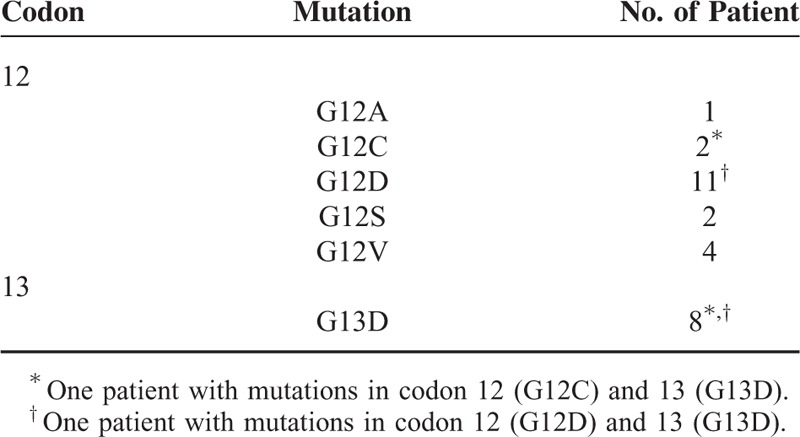
Type of KRAS Mutation (n = 26)

### Pathologic Tumor Response According to the KRAS Mutation Status

After CRT, 28 (28%) of the 100 patients were downstaged from cT3-4N1-2 to ypT0-2N0. Downstaging of the primary tumor was observed in 46 (46%) of the 100 patients, and nodal downstaging was observed in 61 (61%) of the 100 patients. Tumor regression grades after CRT were as follows: 7 patients were grade 4 with total tumor regression, 16 patients with near-total tumor regression (grade 3), 34 patients with moderate regression (grade 2), 42 patients with only minimal regression of the primary tumor (grade 1), and 1 patient with no regression (grade 0).

Tumor response with regard to *KRAS* oncogene status is shown in Table 3. Twenty (27.0%) of the 74 patients in the wild-type KRAS group had downstaged after preoperative CRT. Eight (30.8%) of 26 of the mutant-type KRAS group had downstaged after CRT. There was no statistically significant difference in downstaging rates between the 2 groups (*P* = 0.715). Pathologic T downstaging was achieved in 10 patients (38.5%) of the mutant KRAS group (n = 26) and in 36 patients (48.6%) of the wild-type KRAS group (n = 74). There was no statistically significant difference in T downstaging rates between the 2 groups (*P* = 0.370). Pathologic N downstaging was achieved in 16 patients (61.5%) of the mutant KRAS group (n = 26) and in 45 (60.8%) patients of the wild-type KRAS group (n = 74). Pathologic N downstaging rates were not significantly different between the 2 groups (*P* = 0.948).

**TABLE 3:**
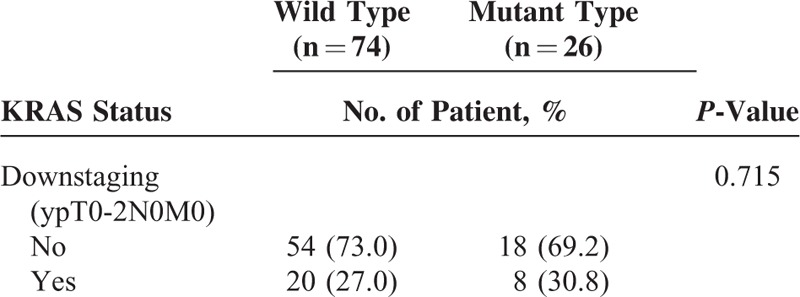
Comparison of Tumor Response With Regard to KRAS Mutation Status

In the multivariate analysis, age, gender, clinical T and classification, tumor grade, location, and size, interval between radiation and operation, *KRAS* oncogene status, and EFGR expression significantly did not affect the tumor response after preoperative CRT. However, CEA level (odds ratio, 3.14; 95% of confidence interval, 1.16–8.45, *P* = 0.033) was a significant factor for downstaging (Table 4).

**TABLE 4:**
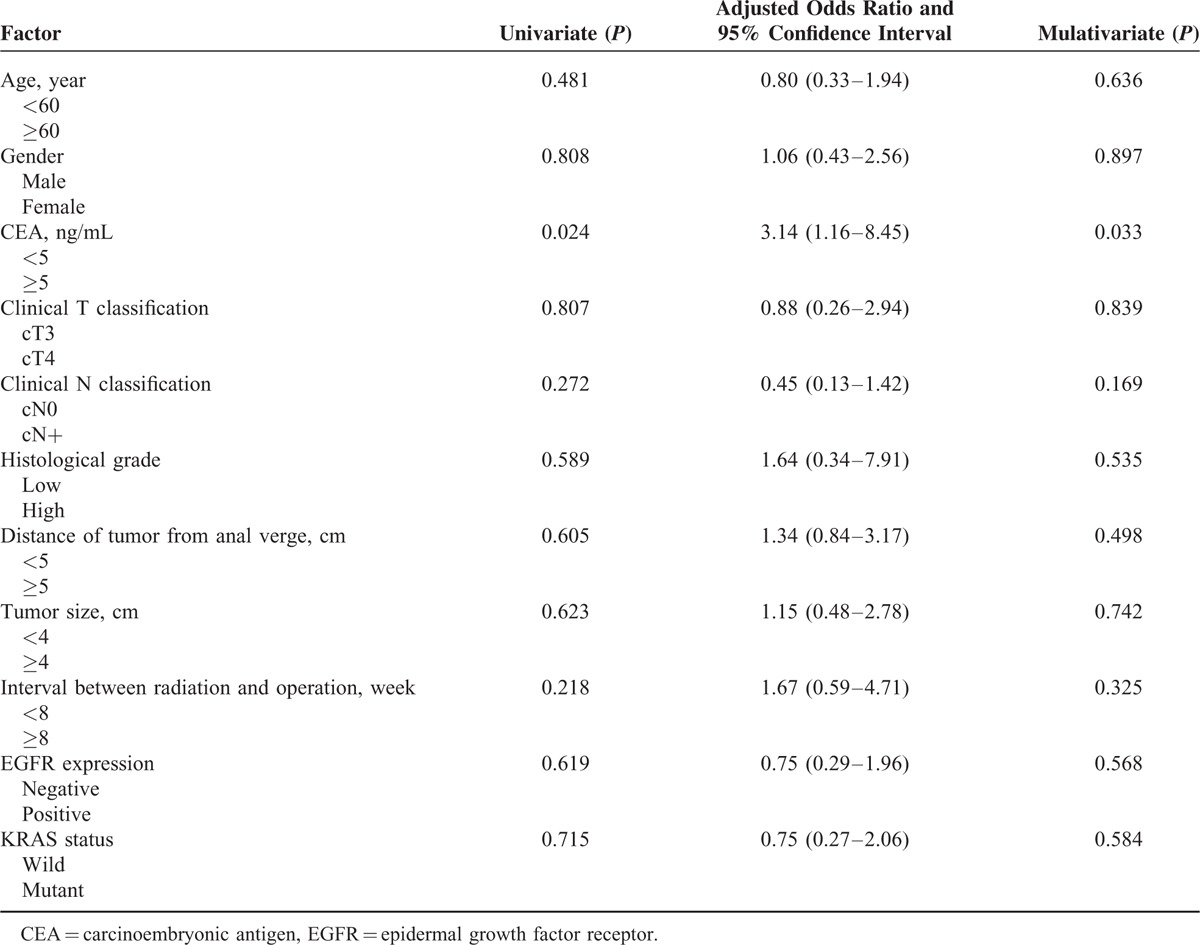
Univariate and Multivariate Analysis of Factors Associated With Tumor Response After Chemoradiotherapy

### Recurrence and Survival According to the KRAS Mutation Status

**FIGURE 2:**
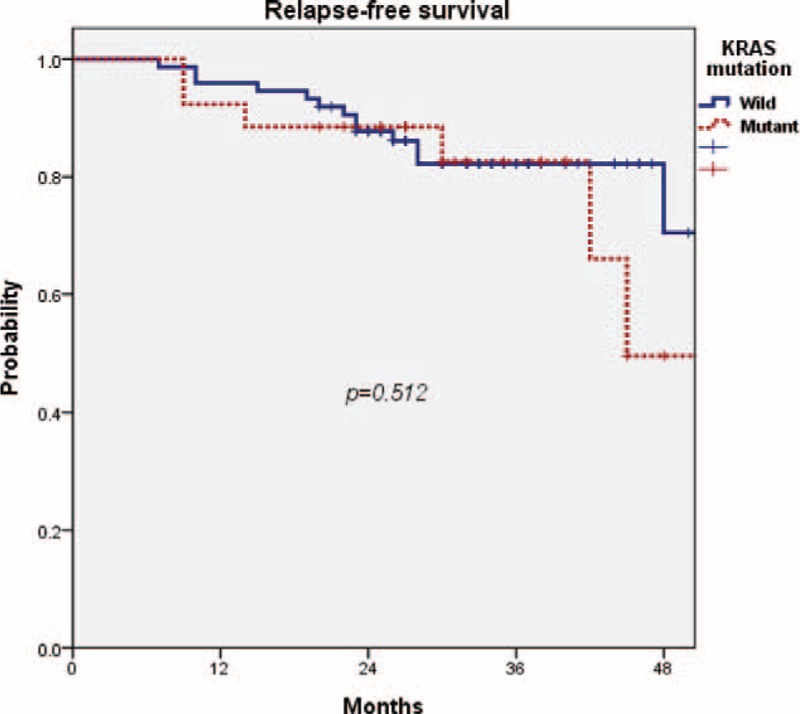
There was no statistically significant difference in the 3-year relapse-free survival rate between the wild-type KRAS group and the mutant-type KRAS group (82.2% vs 82.6%, *P* = 0.512). KRAS = Kirsten rat sarcoma viral oncogene homolog.

**FIGURE 3:**
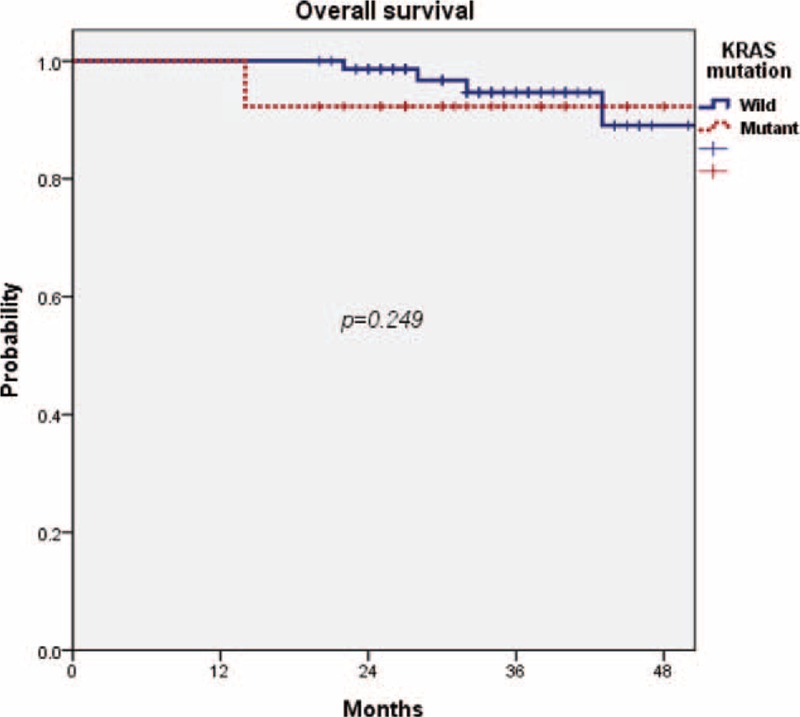
There was no significant difference in the 3-year overall survival rate between the wild-type KRAS group and the mutant type KRAS group (94.7% vs 92.3%, *P* = 0.249). KRAS = Kirsten rat sarcoma viral oncogene homolog.

After a median follow-up time of 34 months (range, 22–140 months), 17 of the 100 patients had recurrent diseases. Locoregional relapse was seen in 6 patients, and distant relapse in 15 patients (lung, 10 cases; liver, 6 cases; and bone, 1 case). Three patients had both locoregional and distant diseases. The RFS rates at 3 years were 82.2% for the wild-type KRAS group and 82.6% for the mutant-type KRAS group. There was no statistically significant difference in the 3-year RFS between the 2 groups (*P* = 0.512) (Figure 2). The OS rates at 3 years were 94.7% for the wild-type KRAS group and 92.3% for the mutant-type KRAS group. There was no significant difference in the 3-year OS rate between the 2 groups (*P* = 0.249) (Figure 3).

## DISCUSSION

The *KRAS* oncogene mutation is involved in the transition of adenoma to carcinoma in colorectal cancer.^20^ Many investigators have studied the *KRAS* gene as a key factor for cancer prognosis, but the prognostic value in colorectal cancer patients is unclear.^18^ EGFR is known to control cell proliferation and apoptosis, cell cycle distribution, and the progression of cancer,^21^ and KRAS is a known transducer in the EGFR pathway.^9^ Several trials examining tumors with KRAS mutation and anti-EGFR therapy have revealed the poor prognostic effect of KRAS mutations in metastatic colorectal cancer patients.^22^

As mentioned earlier, 5-FU or capecitabine-based chemotherapy is the recommended chemotherapy regimen with concurrent radiotherapy before curative surgery. Nevertheless, in this therapeutic setting, clinical studies regarding the *KRAS* oncogene status and treatment outcomes in locally advanced rectal cancer are scarce.^13^ For this reason, we focused on the potential of *KRAS* oncogene status as a biological predictive marker for rectal cancer when 5-FU-based neoadjuvant CRT is conducted in locally advanced rectal cancer.

The extent of response to preoperative CRT significantly affects local and distant metastasis and survival. There are some reports that the good responders after preoperative CRT showed better outcomes than poor responders in the control of local and distant failure.^23^ Thus, tumor response can be an important prognostic factor in rectal cancer. The reported downstaging rate of approximately 30% to 40% has been found in response to 5-FU-based preoperative CRT.^1,23–25^ However, in our analysis, the downstaging (defined as ypT0-2N0M0) rate was 28%. The current study included more patients with clinical T4 tumor stages than previous studies.^1,23–25^ Thus, the current study may have resulted in the relatively lower rate of tumor response than in existing studies. In our series, pretreatment CEA level was the only significant predictor for downstaging in multivariate analysis (odds ratio, 3.14; 95% of confidence interval, 1.16–8.45, *P* = 0.033). Several studies have evaluated the utility of preoperative CEA levels as a predictor of tumor response after preoperative CRT in rectal cancer patients. Lee et al^26^ reported that a preoperative level of CEA >5 ng/mL was associated with a poor tumor response in Asians. Some studies have been reported that rectal cancer with the KRAS mutation is likely to have poor response to preoperative CRT compared with wild-type tumors.^12,27^ However, in the meta-analysis by Clancy et al,^28^ there were no significant differences in tumor response between the wild-type and mutant-type KRAS groups, irrespective of the chemotherapy regimen. In the present study, there was no statistically significant difference in tumor response between the 2 groups.

Several studies analyzed the clinical correlation between KRAS mutation subtypes and treatment outcomes. In the “RASCAL II” study,^20^ the G12V mutation was associated with decreased failure-free survival and OS. Gaedcke el al^8^ reported that tumor regression rate was higher in G12V mutations than in G13D mutations. There were 11 patients with G12D mutation and 8 patients with G13D mutation in the current study. Five of those who had the G12D mutation were downstaged and did not have local or distant failure. None with the G13D mutation was downstaged. Two patients had locoregional failure and 3 patients had distal failure. However, the clinical correlation between KRAS mutation subtypes and treatment outcomes was statistically insignificant in our analysis. The KRAS mutation status is clearly predictive when colorectal cancer patients undergo an anti-EGFR-targeted therapy such as cetumximab.^10^ Since 5-FU and leucovorin obviously do not specifically target the EGFR-signaling pathway, the KRAS mutation status is not a prognostic factor for RFS in our analysis. In a systemic meta-analysis, the presence of KRAS mutation does not affect the cancer-specific survival following neoadjuvant radiotherapy with 5-FU and surgery in rectal cancer.^28^

There are some limitations in our study. First, our study should be understood in view of the inherent biases of a retrospective study design, and we have evaluated small number of the patient cohort. Second, the follow-up duration may have been too short to give a complete description of KRAS mutation and survival. The median follow-up time of the present study is 34 months, and it is shorter than those of other studies.^4,8,29,30^ This may explain the excellent survival outcome regardless of the *KRAS* oncogene status in our study, and longer follow-up would be helpful to elucidate the value of the *KRAS* oncogene and its mutation more clearly.^31^

In our investigational study, we found that the KRAS mutation does not influence the tumor response to preoperative CRT and survival in locally advanced rectal cancer. Prospective trials with longer follow-up examining the biomarkers of rectal cancer will be required for a greater understanding.
